# Skill-Based Verification of Cyber-Physical Systems

**DOI:** 10.1007/978-3-030-45234-6_10

**Published:** 2020-03-13

**Authors:** Alexander Knüppel, Inga Jatzkowski, Marcus Nolte, Thomas Thüm, Tobias Runge, Ina Schaefer

**Affiliations:** 8 University of Paderborn, Paderborn, Germany; 9 ICREA, Open University of Catalonia, Barcelona, Spain; 10 TU Braunschweig, Braunschweig, Germany; 11 University of Ulm, Ulm, Germany

**Keywords:** Deductive verification, design by contract, formal methods, theorem proving, KeYmaera X, hybrid systems, automated reasoning, cyber-physical systems

## Abstract

Cyber-physical systems are ubiquitous nowadays. However, as automation increases, modeling and verifying them becomes increasingly difficult due to the inherently complex physical environment. *Skill graphs* are a means to model complex cyber-physical systems (e.g., vehicle automation systems) by distributing complex behaviors among skills with interfaces between them. We identified that skill graphs have a high potential to be amenable to scalable verification approaches in the early software development process. In this work, we suggest combining skill graphs with hybrid programs. Hybrid programs constitute a program notation for hybrid systems enabling the verification of cyber-physical systems. We provide the first formalization of skill graphs including a notion of compositionality and propose Skeditor, an integrated framework for modeling and verifying them. Skeditor is coupled with the theorem prover KeYmaera X, which is specialized in the verification of hybrid programs. In an experiment exhibiting the *follow mode* of a vehicle, we evaluate our skill-based methodology with respect to savings in verification effort and potential to find modeling defects at design time. Compared to non-compositional verification, the initial verification effort needed is reduced by more than 53%.

### 1 Introduction

*Cyber-physical systems* combine digital computations and physical processes by tightly integrating discrete and continuous dynamics [6]. The last decade has witnessed an increase in the degree of automation in *safety-critical* cyber-physical systems (e.g., such as self-driving cars and transportation in general). Furthermore, the complexity of formally modeling and verifying such systems (e.g., by means of hybrid systems models [11, 19, 30]) to reason about safety increased simultaneously. Although there is a clear desire for an early identification and

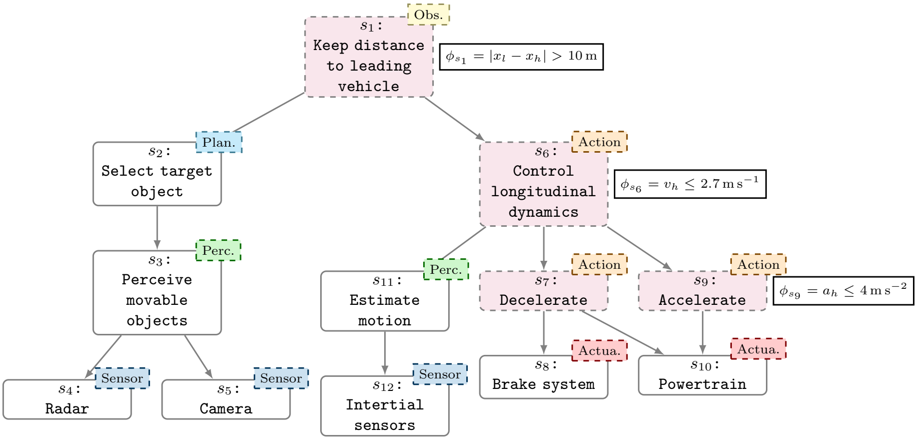

Fig. 1: Excerpt of a skill graph representing an operation to keep distance to a leading vehicle. We illustrate informal safety guarantees for the three skills $s_1$, $s_6$, and $s_9$.

elimination of severe mistakes [9], there is still a remarkable lack of formal methods integrated in the software development cycle [16, 17]. The challenge is to derive modeling and verification approaches that are applicable in the early development stages (e.g., requirements analysis and design time). To address this challenge, we present a model-based verification framework unifying the decomposition and modeling of cyber-physical systems by means of skill graphs [33] and a formal verifcation of these models by means of hybrid systems [2, 3, 5, 25].

A skill is a simple capability (e.g., acceleration in the context of a vehicle) *explicitly* provided by a cyber-physical system. Skills exhibit specific behaviors (i.e., control algorithms) by a mapping to some implementation unit (e.g., *source code* or interacting *software components*). Skills are assigned to a specific category (e.g., *actuator*, *sensor*, or *observable behavior*) with a defined hierarchy to prevent modeling mistakes. This categorization follows the design principle of *separation of concerns* [27], which ensures that skills only have single well-defined responsibilities. Separation of concerns is known to have a positive effect on modeling complexity, comprehensibility, functional reusability, fault localization, and artifact traceability [15, 36]. Skills can be annotated with safety guarantees obtained from a preceding requirements analysis, which enables the application of verification techniques.

Skill graphs, informally introduced by Reschka et al. [32, 33], are a promising means to model complex actions of cyber-physical systems from an architectural point of view. A skill graph [26, 32, 33, 37] is a directed acyclic graph comprising a set of *skills* (i.e., nodes) and dependencies between them (i.e., edges). To describe the properties we want to verify in a skill graph, we illustrate a skill graph representing a driving task in Figure 1. The task exhibits that a vehicle autonomously tries to keep a distance of at least 10 m to a leading vehicle. On the top level, skill ***Keep distance to leading vehicle*** ($s_1$) depends on two other skills, namely (1) the planning skill ***Select target object*** ($s_2$) and (2) the action skill ***Control the longitudinal dynamics*** ($s_6$). Whereas sensor-dependent skills are typically realized by software algorithms only (e.g., deep learning for detecting an obstacle), actuator-dependent skills (highlighted with a dashed border) also need to incorporate control theory, as the physical environment has to be taken into account. Skills are annotated with safety requirements (e.g., maximum acceleration or minimum distance to other vehicles). Together with the skill's realization and its dependencies to other skills, this requirement expresses the property we want to verify at design time. Successfully verifying all skills in the context of a skill graph ensures that the represented task complies to the complete set of safety requirements.

Conceptually, skill graphs as applied in this work are used for designing and organizing the architecture of a cyber-physical system. First, they facilitate the modeling of complex maneuvers built from simpler skills, which interact through explicit *interfaces*. Second, they advocate the systematic reuse of ready-to-integrate skills for multiple skill graphs, which reduces maintenance costs and increases software quality in general. Third, skill graphs are intuitive and therefore accommodate good potential for communicating with stakeholders and non-experts. Typically, skill graphs are supplied with performance measurements with the goal to enforce safety requirements at run-time. We are the first to exploit skill graphs to formally reason about the satisfiability of safety requirements at design time. Both areas of application complement each other, as they cover the full range from static analysis in the design phase to run-time verification and monitoring during operation.

As the foundation for our model-based verification approach, we propose to realize skills that interact with the physical environment by means of hybrid systems based on the *differential dynamic logic* $d\mathcal{L}$ [28, 29, 31]. Hybrid systems represent complex physical systems, typically modeled as automata, where states are defined by continuous variables based on differential equations and transitions between states are discrete. Differential dynamic logic enables the deductive verification of hybrid systems, and as such is suitable for reasoning automatically about the correctness of hybrid systems. The key step of our approach is to decompose complex tasks of a cyber-physical system into skills connected by means of a skill graph and to provide a translation of skills to hybrid systems. The combination of skill graphs and hybrid systems allows the identification of severe mistakes during early design phases and also – in case of success – to generate correctness proofs, which increases trust that the system under design behaves as intended. Moreover, we propose a notion of compositionality for skill graphs, which is crucial to manage scalability during the verification phase. While skill graphs may only model simple functional aspects, they can be assembled to exhibit more complex behaviors, and verification results of skills can be reused.

We have implemented a prototype for modeling and verifying skill graphs called SKEDITOR. SKEDITOR supports the graphical modeling of skill graphs, allows to specify safety guarantees, and enables formal verification through a mapping to hybrid programs [30] (i.e., a program notation for hybrid systems as required by the theorem prover KEYMAERA X [14]). In a case study exhibiting

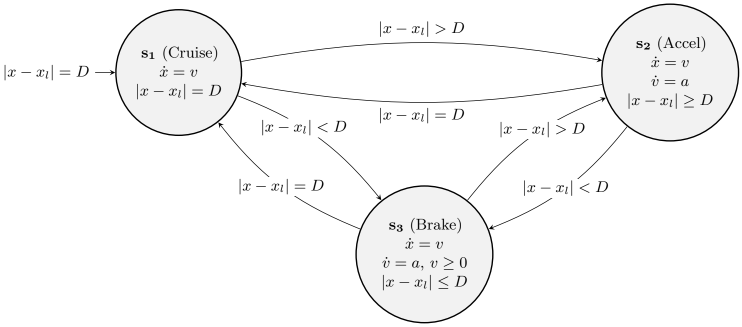

Fig. 2: Simplified hybrid system of a vehicle with automatic headway control.

the *follow mode* of an automated vehicle, we evaluate SKEDITOR with respect to its potential to find modeling defects. In particular, SKEDITOR allowed us to find conceptual defects of control algorithms early on in the design phase of our case study. To summarize, the contribution of this work is threefold.

- **Framework:** We are the first to formalize skill graphs and propose *skill-based verification*, a model-based verification technique allowing us to identify poorly defined safety requirements in early design phases by combining skill graphs with hybrid programs.
- **Tool support:** We implemented skill-based verification in a prototypical open-source tool called SKEDITOR, which paves the way for users to model and verify cyber-physical systems based on skill graphs.
- **Evaluation:** We demonstrate our approach on a realistic case study examining the *follow mode* of an automated vehicle. We show that skill-based verification decreases effort compared to monolithic modeling.

### 2 Background on Hybrid-System Modeling

A prominent mathematical foundation for cyber-physical systems is constituted by *hybrid systems* [2, 11, 19, 30], which enable a mixed modeling of continuous dynamics (expressed by differential equations) and discrete dynamics (expressed by automata). The states change on the basis of flow conditions.

**Example 1** *Consider the example of an automatic headway control of a vehicle depicted in Figure 2. Four variables exist: the host's current position ($x$), the current position of the leading vehicle ($x_l$), the current velocity ($v$), and the current acceleration ($a$). The headway control exhibits three states: ($\mathbf{s_1}$) the vehicle is in cruise mode when the current distance to the leading vehicle is equal to a defined constant $D$, ($\mathbf{s_2}$) the vehicle accelerates when the distance is greater than $D$, and ($\mathbf{s_3}$) the vehicle decelerates when the distance is less than $D$, but only until the vehicle comes to a full stop. The headway control ensures that the distance to the leading vehicle is approximately equal to $D$.*

*Hybrid programs* define an imperative-like program notation for hybrid systems [28], which support the definition of variables that evolve along a differential equation and are interpreted by tools such as KeYmaera X [14]. The syntax of hybrid programs is as follows.

$$\alpha ::= \alpha; \beta \mid \alpha \cup \beta \mid \alpha^* \mid x := \Theta \mid x := * \mid x' = \Theta \,\&\, H \mid ?H \tag{1}$$

$\alpha; \beta$ represents the sequential composition of two hybrid programs. $\alpha \cup \beta$ expresses the non-deterministic choice between two hybrid programs. $\alpha^*$ expresses that the execution of $\alpha$ may be repeated zero or more times. The discrete assignment to $x$ is either a term $\Theta$ (possibly over $x$) or an arbitrary value represented by the wildcard $*$. The continuous evolution of a variable $x$ along a differential equation is described by $x' = \Theta \,\&\, H$, where $H$ is an optional evolution domain. Finally, $?H$ describes a testable condition that aborts the evolution if $H$ is false. For instance, the program $\alpha \doteq (v := *; a := *;\ ?(-a_b \leq a \leq 0); \{v' = a \,\&\, v \geq 0\})$ sets velocity $v$ to an arbitrary value and acceleration $a$ to a value between $-a_b$ (i.e., maximum braking force) and zero. The execution stops nondeterministically at any time but at the latest before velocity $v$ reaches a negative value.

Semantics of hybrid programs are based on differential dynamic logic $d\mathcal{L}$ [28, 29, 31] to specify and verify properties of hybrid programs associated with a skill in a skill graph. Models specified in $d\mathcal{L}$ can be verified with KeYmaera X, a matured open-source theorem prover for hybrid programs. The following grammar describes all valid formulas of $d\mathcal{L}$. Symbol $\sim$ is a placeholder for a comparison operator (i.e., $\sim \in \{<, \leq, >, \geq, =, \neq\}$) between two terms $\Theta_1$ and $\Theta_2$. Terms are polynomials with rational coefficients over the set of continuous variables.

$$\Phi ::= \Theta_1 \sim \Theta_2 \mid \neg\Phi \mid \Phi \wedge \Psi \mid \Phi \vee \Psi \mid \Phi \rightarrow \Psi \mid \forall x\, \Phi \mid \exists x\, \Phi \mid [\alpha]\Phi \tag{2}$$

The semantics of the logical connectives is defined as in first-order logic. Additionally, the modal formula $[\alpha]\Phi$ holds if all runs of the hybrid program $\alpha$ end in a state that satisfies the given condition $\Phi$. Following the idea of Hoare-style specification in classical deductive reasoning [1, 7, 10, 18, 34], we are particularly interested to prove validity of the condition $\Psi \rightarrow [\alpha]\Phi$ with $\Psi$ expressing assumptions we have and $\Phi$ expressing guarantees to meet by the hybrid program $\alpha$.

### 3 A Formalization for Skill-Based Modeling

In this section, we propose the first formalization of modeling cyber-physical systems based on skill graphs. First, we define the essence of a skill. Second, we continue with the definition of a skill graph and what makes it well-formed. Third, we define how to compose skill graphs to exhibit more complex behaviors.

#### 3.1 Formalizing Skills

In the context of cyber-physical systems, *skills* describe fine-grained executable activities inspired by human behaviors [32, 33]. For instance, a skill may represent longitudinal driving (i.e., driving with constant velocity) or even a more complex combination of longitudinal and lateral maneuvers (i.e., following the lane). To ensure that such maneuvers are executed safely, skills are associated with so-called *safety guarantees*, which they must fulfill to be considered *safe*. For example, a skill exhibiting the following of a leading vehicle should keep a minimum distance of a specified constant $D$ (cf. Fig. 2). Informal safety guarantees are typically formulated by experts who identify numerous hazardous scenarios with respect to a maneuver and resolutions to prevent them.

The implementation of skills was only vaguely specified before. Typically, skills are implemented by software components [33]. However, our goal of early verification at design time requires to also consider a model of the physical environment. Therefore, we propose to implement skills by hybrid programs [28], which already incorporate assumptions about the physical environment and enable the verification of implementation against safety guarantees *at design time*.

To separate concerns, a skill has an associated type. We define the set $\texttt{Type} = \{\texttt{observable behavior}, \texttt{action}, \texttt{perception}, \texttt{planning}, \texttt{sensor}, \texttt{actuator}\}$, which categorizes the purpose of a skill. Moreover, a skill has dependency-relationships with other skills. Informally, the idea is that a hybrid program of a skill may introduce a set of continuous state variables, their computation, and their valid domains (e.g., velocity $v \in [0, 60]$ with $v' = a$), but may also require the presence of variables and their domains defined by other skills (e.g., acceleration $a \in [0, 4]$). In the following, we formally define a skill. Let $\mathcal{X}$ denote the universe of continuous variables. The syntactic domain of a skill is defined as follows.

**Definition 1 (Skill).** *A* skill *is a 5-tuple* $\langle X_{\texttt{def}}, X_{\texttt{req}}, \alpha, \tau, \Phi \rangle$, *where*

- $X_{\texttt{def}} \subseteq \mathcal{X}$ *is a finite set of variables defined in the hybrid program* $\alpha$,
- $X_{\texttt{req}} \subseteq \mathcal{X}$ *is a finite set of variables required by the hybrid program* $\alpha$,
- $\alpha$ *is the (possibly empty) hybrid program (cf. Eq. 1) over variables in* $X_{\texttt{def}} \cup X_{\texttt{req}}$,
- $\tau \in \texttt{Type}$ *is the associated type,*
- $\Phi = \{\phi_1, \ldots, \phi_m\}$ *is a finite set of safety guarantees in first-order logic over variables in* $X_{\texttt{def}} \cup X_{\texttt{req}}$ *(cf. Eq. 2).*

*To be well-formed, we require that the sets of defined and required variables of a skill are disjoint (i.e.,* $X_{\texttt{def}} \cap X_{\texttt{req}} = \emptyset$*). To access a skill's attribute, we use the '*`.`*' (dot) operator (e.g.,* $s.\tau$ *expresses the type of skill* $s$*).*

#### 3.2 Formalizing Skill Graphs

We formalize skill graphs as directed acyclic graphs comprising a set of skills (i.e., nodes), which are connected through directed edges representing their dependencies. We denote by $\mathcal{S}$ the universe of all skills and define the syntactic domain of skill graphs as follows.

**Definition 2 (Skill Graph).** *A* skill graph *is given by* $G \mathrel{\hat{=}} \langle S, r, E \rangle$, *where*

- $S \subset \mathcal{S}$ *is a finite set of skills,*
- $r \in S$ *is the root skill,*
- $E \subseteq S \times S$ *is set of directed edges between skills. We denote* $(s_c, s_p) \in E$ *as* $s_c \prec s_p$ *meaning that* $s_c$ *is a child of* $s_p$.

| $\tau_s \setminus \tau_t$ | observable | action | actuator | planning | perception | sensor |
|---|---|---|---|---|---|---|
| observable | ✓ | ✓ | - | ✓ | ✓ | - |
| action | - | ✓ | ✓ | ✓ | ✓ | - |
| planning | - | - | - | ✓ | ✓ | - |
| perception | - | - | - | - | ✓ | ✓ |

Table 1: Valid types of a child skill $t$ for a skill $s$ (i.e., $t \prec s$).

A skill graph is an acyclic directed graph with exactly one root skill $r$. To guarantee that skill graphs are *well-formed*, we impose specific constraints. We formally introduce the *path* between two skills as follows.

**Definition 3 (Path).** *Let $E$ be a set of edges and $s_1, \ldots, s_l \in S$ skills of a skill graph. A* path *of length $l-1$ is a (possibly empty) sequence of $l-1$ edges $(s_1, s_2)$, $(s_2, s_3)$, ..., $(s_{l-1}, s_l) \in E$ denoted by $\pi_{s_1 \to s_l} = [(s_1, s_2), (s_2, s_3), \ldots, (s_{l-1}, s_l)]$. We say that a path between skills $s, s' \in S$* exists *if $\pi_{s \to s'}$ is non-empty, and does* not exists *otherwise.*

As mentioned before, each skill has an assigned type. Based on our definition of a well-formed graph, we enforce that only skills with particular types can form valid parent-child relationships (cf. Table 1). For instance, for two skills $s, s' \in S$, if $s \prec s'$ holds and skill $s'$ is of type `perception`, then skill $s$ is only allowed to have type `sensor` or `perception`.

**Definition 4 (Well-Formed Skill Graph).** *Let $G = \langle S, r, E \rangle$ be a skill graph. $G$ is* well-formed *if and only if*

- *each skill $s \in S \setminus \{r\}$ in a skill graph has at least one parent skill $s' \in S$ (i.e., $\{s' \in S \mid s \prec s'\} \neq \emptyset$) and there exists at least one path from skill $s$ to root skill $r$,*
- *for each edge $(s, s') \in E$, skills $s, s'$ satisfy the typing restriction depicted in Table 1,*
- *for each skill $s \in S$ and variable $x \in s.X_{\texttt{req}}$ there exists a path $\pi_{s' \to s'}$ from a skill $s' \in S$ that introduces variable $x$ (i.e., $x \in s'.X_{\texttt{def}}$),*
- *for each pair of skills $s, s' \in S$, the sets of defined variables are disjoint (i.e., $s.X_{\texttt{def}} \cap s'.X_{\texttt{def}} = \emptyset$).*
- *for each skill $s$ in $G$, formula $\bigwedge_{\phi \in s'.\Phi \wedge s' \prec s} \phi$ must be satisfiable.*

*Remark.* Unlike behavioral models, skill graphs as defined here do not suggest an execution order of skills on the same level (i.e., child skills). The reason is twofold. First, the information needed for the scheduling may be incomplete at design time (i.e., concrete hardware and scheduling parameters). Second, the intent of skill graphs is to abstract away from implementation details, while providing guarantees about the correctness of defined safety requirements. In Section 4.2, we illustrate how to assemble the decomposed hybrid programs of a skill graph to a complete hybrid program, while being safe with respect to our chosen level of abstraction.

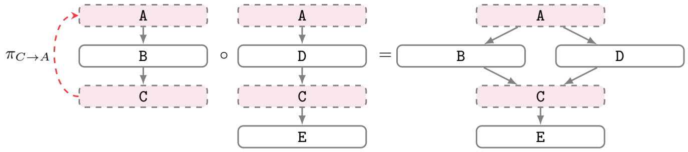

Fig. 3: Example of a composition of two skill graphs.

#### 3.3 Composition of Skill Graphs

From the perspective of software engineering practices, an advantage of skill graphs is their modular nature. Multiple skill graphs can be designed in isolation, but may also share the same skills. To model and verify more complex skill graphs and to prevent unnecessary redundancy, the idea is to adequately *reuse* previously designed skill graphs and subsequently compose them together. This method further supports the identification and location of design mistakes, maintenance of skill graphs in general, and also enables the distribution of modeling tasks in multi-team software development.

Our composition technique of skill graphs is inspired by *superimposition* [8], a simple process that composes two graphs recursively together by merging their substructures. Starting from the root skill of one of the skill graphs, skills at the same level fulfilling defined criteria can then be composed to form a new resulting skill. Starting from a joint root skill of two different skill graphs $G_1 = \langle S_1, r, E_1 \rangle$ and $G_2 = \langle S_2, r, E_2 \rangle$, two skills $s_1 \in S_1$ and $s_2 \in S_2$ are composed to a new skill $s$ if:

– both paths, $\pi_{s_1 \to s_1'}$ and $\pi_{s_2 \to s_2'}$, exist and $s_1'$, $s_2'$ are already composed,
– $s_1$ and $s_2$ have an equal type and equal sets of defined and required variables,
– and either any of the two hybrid programs is empty or both are identical.

For illustration, Fig. 3 depicts an abstraction of the composition of two skill graphs. Both skill graphs share the identical skills $A$ and $C$. First, the root skill $A$ of both skill graphs is superimposed, and second, skill $C$ is superimposed after identifying that in both skill graphs there exists a path to a skill already subject to composition (i.e., $A$). In the following, we call two skills from different skill graphs *composable* if they are subject to the composition as explained here. The resulting skill $s$ receives all the properties (i.e., variables, type, and hybrid program) from the composable skills and additionally the union of their safety guarantees:

**Definition 5 (Composition of Skills).** *Let $s_1 \in S_1$ and $s_2 \in S_2$ be two composable skills. The binary* composition *of $s_1$ and $s_2$ then produces the skill*

$$s_1 \oplus s_2 = \left\langle s_1.X_{\texttt{def}}, s_1.X_{\texttt{req}}, s_1.\alpha, s_1.\tau, s_1.\Phi \cup s_2.\Phi \right\rangle . \quad (3)$$

The binary composition of two skill graphs is then formally defined as follows, where $M = \{(s_1, s_2) \in S_1 \times S_2 \,|\, s_1 \text{ and } s_2 \text{ are composable}\}$ is the set of composable skills and $f$ is a function that maps every skill in $(S_1 \cup S_2) \setminus \{s_1, s_2 \in S_1 \cup S_2 \,|\, (s_1, s_2) \in M\}$ to itself and maps all skills $s_1, s_2$ with $(s_1, s_2) \in M$ to a new skill $s = s_1 \oplus s_2$.

**Definition 6 (Composition of Skill Graphs).** *Let* $G_1 = \langle S_1, r_1, E_1 \rangle$ *and* $G_2 = \langle S_2, r_2, E_2 \rangle$ *be two well-formed skill graphs with* $r_2 \in S_1$*. The composition of* $G_1$ *and* $G_2$ *then produces the skill graph*

$$G_1 \circ G_2 = \langle S, f(r_1), E \rangle \tag{4}$$

*where*

- $S = \{f(s) \mid s \in S_1 \cup S_2\}$,
- *for every* $s, s' \in (S_1 \cup S_2)$*, there exists an edge* $(f(s), f(s')) \in E$ *if and only if there exists an edge* $(s, s') \in (E_1 \cup E_2)$*.*

A mathematical convenience of our definition of composition is that it requires the root skill of one skill graph to be present in the second skill graph. This is not a severe limitation, as it is always possible to add an artificial root to one skill graph (or both) with respect to well-formedness.

### 4 Compositional Verification of Skill Graphs

In this section, we formalize the generation of verification conditions to check correctness of skills in the context of a skill graph, show how correctness results transfer to the composition of skill graphs, and discuss how this methodology can be integrated into the development process for cyber-physical systems.

#### 4.1 Verification Condition Generation

Our verification procedure relies on assume-guarantee reasoning. Thus, to verify whether a skill $s$ in the context of a skill graph adheres to its safety guarantees $s.\Phi$, we have to construct two logical conditions: (1) necessary assumptions on a skill's behavior denoted by $\texttt{assume}_s$ and (2) the overall safety condition in the context of the skill graph denoted by $\texttt{safe}_s$. For instance, $\texttt{assume}_s$ for leaf skills valuates trivially to `true`, but child skills impose constraints on their parent skills through their safety guarantees. Both conditions can be computed automatically based on the skill's dependencies and by the manually defined safety guarantees $s.\Phi$. The overall verification condition then becomes $\texttt{assume}_s \rightarrow [s.\alpha]\texttt{safe}_s$ (cf. Sec. 2). In the following, we describe how both conditions are constructed.

In the context of a skill graph, a particularity to deal with is that a skill may *require* variables introduced in a *distant* skill (i.e., path length greater than one), possibly with numerous updates along the path. These variables may be unknown in direct children, so it is not possible to only define the assumption (i.e., $\texttt{assume}_s$) of a skill $s$ as the conjunction of the safety guarantees of all children (i.e., $\bigwedge \texttt{safe}_{s'}$ with $s' \prec s$). In Fig. 4, we illustrate this problem and its solution on a simple skill graph comprising three skills.

`Skill #1` introduces variables `A` and `B` including safety guarantees on them in $\phi_1$. Typical for assume-guarantee reasoning, $\phi_1$ becomes the assumption for all parent skills (i.e., `Skill #2` in this case). However, the safety guarantee of `Skill #2` (i.e., $\phi_2$) states only a modification of variable $A$ and not $B$, but `Skill`

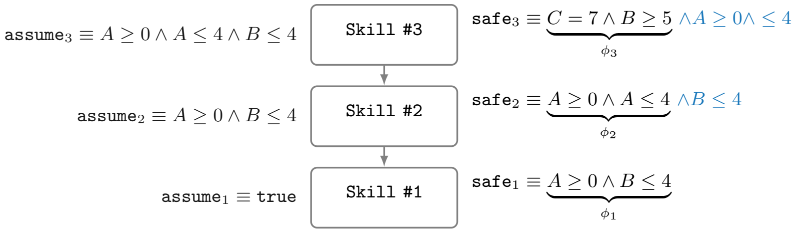

Fig. 4: Computation of $\texttt{assume}_t$ and $\texttt{safe}_t$.

##3 may indeed need the information of the current domain of variable $B$ to be verifiable. To keep assume-guarantee propagation intact, we resolve this issue by additionally encoding all safety guarantees that *remain valid* for a skill in its safety guarantee $\texttt{safe}_s$ (highlighted in blue). In the following, we introduce our formalization.

The definitions of both formulas, $\texttt{assume}_s$ and $\texttt{safe}_s$, are mutually recursive. The logical formula $\texttt{assume}_s$ for a skill $s$ results from the conjunction of the overall safety guarantees $\texttt{safe}_{s'}$ of all children $s' \prec s$. The assumption for skills with no children valuates trivially to $\texttt{true}$.

$$\texttt{assume}_s \equiv \bigwedge_{s' \prec s} \texttt{safe}_{s'} \tag{5}$$

To compute the overall safety guarantee $\texttt{safe}_s$, we exploit that $\texttt{assume}_s$ exhibits an overapproximation on the current state of the required variables for a skill $s$ prior to executing the hybrid program $s.\alpha$. As the behavior of a skill may change the initial state, we discard all clauses in $\texttt{assume}_s$ sharing a variable with one of the user provided safety guarantees in $s.\varPhi$. The remaining clauses become part of $\texttt{safe}_s$. For instance, in Fig. 4, Skill #3 guarantees a change of variable $B$ in $\phi_1$. Thus, only clauses of $\texttt{assume}_3$ without mentioning $B$ transfer to $\texttt{safe}_3$. For mathematical convenience, we denote the conjunction of all safety guarantees of a skill by the logical formula $\phi_s \equiv \bigwedge_{\phi \in s.\varPhi} \phi$ and the set of assumptions of a skill in a skill graph by the set $\mathcal{A}_s = \{\psi_1, \ldots, \psi_n \mid \texttt{assume}_s \equiv \psi_1 \wedge \cdots \wedge \psi_n\}$. Furthermore, set $\texttt{var}(\cdot)$ denotes the set of variables of a logical formula. The overall safety guarantee of a skill is then computed as follows.

$$\texttt{safe}_s \equiv \phi_s \wedge ( \bigwedge_{\substack{\psi \in \mathcal{A}_s \wedge \\ \texttt{var}(\phi_s) \cap \texttt{var}(\psi) = \emptyset}} \psi) \tag{6}$$

We can now define the *validity* of a skill graph as follows.

**Definition 7 (Valid Skill Graph).** *Let $G = \langle S, r, E\rangle$ be a well-formed skill graph. We say that skill graph $G$ is* valid *if and only if $\forall s \in S$ formula $\texttt{assume}_s$ is satisfiable and formula $\texttt{assume}_s \rightarrow [s.\alpha]\texttt{safe}_s$ is valid. We denote by $s \models_G s.\varPhi$ the* validity *of a skill $s$ in a skill graph $G$ with respect to its safety guarantees and by $\models G$ the* validity *of the entire skill graph (i.e., $\models G \equiv \forall s \in S, s \models_G s.\varPhi$).*

The upcoming important theorem states that the individual validity of two skill graphs also transfers to the validity of their composition. However, based on

Def. 6, composition may also lead to an invalid skill graph if the assumption of a skill in the new skill graph is not satisfiable (e.g., possible in case of diamond structures). Therefore, we require satisfiability checks for the computed assumptions and define the *compatibility* between two skill graphs as follows.

**Definition 8 (Compatible Skill Graphs).** *Let $G_1$ and $G_2$ be two well-formed skill graphs. We say that $G_1$ and $G_2$ are* compatible *if the following holds.*

- *$G_1 \circ G_2$ is a well-formed skill graph,*
- *for each skill $s$ in $G_1 \circ G_2$, formula $\texttt{assume}_s$ is satisfiable.*

**Theorem 1 (Composition of Skill Graphs Retains Validity).** *Let $G_1$ and $G_2$ be two compatible skill graphs and $G = G_1 \circ G_2$ their composition. Then, $G$ is valid if $G_1$ and $G_2$ are valid (i.e., $\models G$ if $\models G_1$ and $\models G_2$).*

*Proof.* Let $s_1$ and $s_2$ be two composed skills and $s = s_1 \oplus s_2$ their composition. Following Def. 6, the verification condition for $s$ becomes

$$(\texttt{assume}_{s_1} \land \texttt{assume}_{s_2}) \rightarrow [s.\alpha](\texttt{safe}_{s_1} \land \texttt{safe}_{s_2}).$$

Based on the semantics of $d\mathcal{L}$ [31], condition $\Psi \rightarrow [\alpha]\Phi_1 \land \Psi \rightarrow [\alpha]\Phi_2 \leftrightarrow \Psi \rightarrow [\alpha](\Phi_1 \land \Phi_2)$ holds. As the hybrid programs of $s_1$ and $s_2$ are identical (or at least one of them is empty), the resulting two conditions to check are the following:

$$(1) \qquad (\texttt{assume}_{s_1} \land \texttt{assume}_{s_2}) \rightarrow [s_1.\alpha](\texttt{safe}_{s_1})$$
$$(2) \qquad (\texttt{assume}_{s_1} \land \texttt{assume}_{s_2}) \rightarrow [s_2.\alpha](\texttt{safe}_{s_2})$$

Satisfiability of $(\texttt{assume}_{s_1} \land \texttt{assume}_{s_2})$ follows from Def. 8. Then, validity of both conditions follow from Def. 7 and, consequently, $\models G$ holds. □

### 4.2 Assembling Hybrid Programs in a Skill graph

Skill graphs decompose the system into smaller parts. Likewise, the hybrid program that represents the complete behavior is also distributed over the skill graph. Now that we have defined the structure and behavior of single skills in the context of a skill graph, we define how we can construct the complete behavior of a skill as a single monolithic hybrid program. The resulting hybrid program is then a complete representation of the skill's behavior while also retaining all safety guarantees without the need of re-verifying skills or even entire skill graphs. We start by giving a definition on *how* hybrid programs of skills are assembled together.

**Definition 9 (Hybrid Program Assembly).** *Let $G = \langle S, r, E\rangle$ be a skill graph, $HP$ the set of all hybrid programs, and let $s \in S$ denote an arbitrary skill of $G$. A* hybrid program assembly *of $s$ is a function $\rho : S \rightarrow HP$, which is recursively defined as follows.*

$$\rho(s) = \begin{cases} s.\alpha & \text{if } s \text{ has no children (i.e., } \neg\exists s' \in S : s' \prec s) \\ (\bigcup_{s' \prec s} \rho(s')); s.\alpha & \text{otherwise} \end{cases}$$

The motivation is that such assemblies are safe to be used in other contexts, such as code generation for the validation of prototypes or monitor generation. Assuming a valid skill graph $G$, the following theorem guarantees that any hybrid program assembly over skills in $G$ retains the respective safety guarantees.

**Theorem 2 (Safety Compliance of Hybrid Program Assemblies).** *Let $G = \langle S, r, E\rangle$ be a valid skill graph and let $s \in S$ denote an arbitrary skill of $G$. Then, formula $[\rho(s)]\texttt{safe}_s$ is valid.*

*Proof.* We proceed by induction on the skills of skill graph $G$. For the basis step, we assume that $s$ has no children (i.e., $\neg\exists s' \in S : s' \prec s$). Because $[\rho(s)]\texttt{safe}_s \equiv [s.\alpha]\texttt{safe}_s$ and $G$ is a valid skill graph, it follows from Def. 7 that formula $[\rho(s)]\texttt{safe}_s$ is valid. From now on, we assume that $s$ has children. Our induction hypothesis is that if for each skill $s' \prec s$ program assembly $\rho(s')$ satisfies $\texttt{safe}_{s'}$, then hybrid program assembly $\rho(s)$ satisfies $\texttt{safe}_s$:

$$\begin{array}{lll}
\textbf{(IH)} & & \Big(\bigwedge_{s'\prec s} [\rho(s')]\texttt{safe}_{s'}\Big) \rightarrow [\rho(s)]\texttt{safe}_s \\
(1) & \leftrightarrow & \Big(\bigwedge_{s'\prec s} [\rho(s')]\texttt{safe}_{s'}\Big) \rightarrow \Big[\bigcup_{s'\prec s} \rho(s'); s.\alpha\Big]\texttt{safe}_s \\
(2) & \leftrightarrow & \Big(\bigwedge_{s'\prec s} [\rho(s')]\texttt{safe}_{s'}\Big) \rightarrow \Big[\bigcup_{s'\prec s} \rho(s')\Big][s.\alpha]\texttt{safe}_s \\
(3) & \leftrightarrow & \Big(\bigwedge_{s'\prec s} [\rho(s')]\texttt{safe}_{s'}\Big) \rightarrow \bigwedge_{s'\prec s} [\rho(s')][s.\alpha]\texttt{safe}_s \\
(4) & \leftrightarrow & \Big(\bigwedge_{s'\prec s} \texttt{safe}_{s'}\Big) \rightarrow [s.\alpha]\texttt{safe}_s \\
(5) & \leftrightarrow & \texttt{assume}_s \rightarrow [s.\alpha]\texttt{safe}_s
\end{array}$$

Transformation step (1) follows from substituting $\rho(s)$ with its definition given in Def. 9. Steps (2)–(4) are again based on the semantics of $d\mathcal{L}$ [31]. Step (2) follows from the *sequential composition axiom* $[a; b]P \leftrightarrow [a][b]P$, step (3) from the *nondeterministic choice axiom* $[a \cup b]P \leftrightarrow [a]P \wedge [b]P$, and step (4) from *monotonicity*. Because $G$ is a valid skill graph, validity of $\texttt{assume}_s \rightarrow [s.\alpha]\texttt{safe}_s$ follows again from Def. 7. Consequently, $[\rho(s)]\texttt{safe}_s$ is valid. □

#### 4.3 Integration into the Software Development Process

In Figure 5, we summarize the methodology for modeling and verifying skill graphs. The main idea is that the safety verification of skill graphs modeled in isolation transfers to the composition of compatible skill graphs. This (a) eases the modeling process, as smaller models tend to be less complex and easier to repair, (b) fosters reusability, which is known to be cost-effective and less error-prone, and (c) is promising for scaling the verification to large skill graphs.

In particular, the methodology consists of five major parts. In the first part (*1*), practitioners define and model skills together with their hybrid programs and relevant safety guarantees in isolation and subsequently connect them to form well-formed skill graphs (if possible). In the second part (*2*), for each

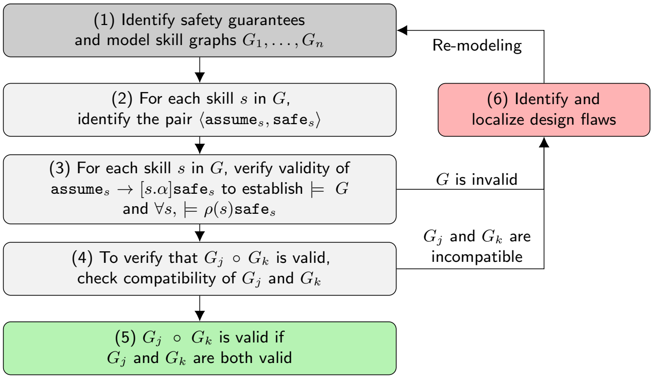

Fig. 5: Methodology of modeling and verifying skill graphs.

skill $s$ in a skill graph, the assumption $\texttt{assume}_s$ and safety guarantee $\texttt{safe}_s$ are computed by evaluating the context of the skill in the skill graph. The third part *(3)* uses the identified assumptions and the safety guarantee to validate each skill in a skill graph individually. If each skill is proven valid (cf. Theorem 1), the complete skill graph is proven valid and can be put into a repository to be reused. Following Theorem 2, all program assemblies over skills in this skill graph retain the respective safety guarantees. The fourth part *(4)* becomes relevant, if two skill graphs are composed together to represent a more complex task of a cyber-physical system. In this case, compatibility of the skill graphs is checked and, if successful, the validity of the composed skill graph is established *(5)*. The final part *(6)* is relevant in the presence of unsuccessful proof attempts. If validity of a skill graph or the composition of multiple skill graphs cannot be established, practitioners need to identify and fix mistakes in their models. Typically, the complexity of localizing design mistakes is reduced with our methodology, as it is explicitly known which exact skills in a skill graph with respect to their safety guarantees could not be verified.

### 5 Evaluation and Discussion

We evaluate our skill-based verification approach on a case study to answer the following two research questions.

**RQ-1** *How does the skill-based methodology compare to monolithic modeling and verification?*

**RQ-2** *To what extent can skill-based compositional verification reduce the verification effort?*

#### 5.1 Open-Source Implementation

We implemented skill-based verification in a tool with the name Skeditor. The implementation is written in Java as an Eclipse plug-in based on Graphiti [13],

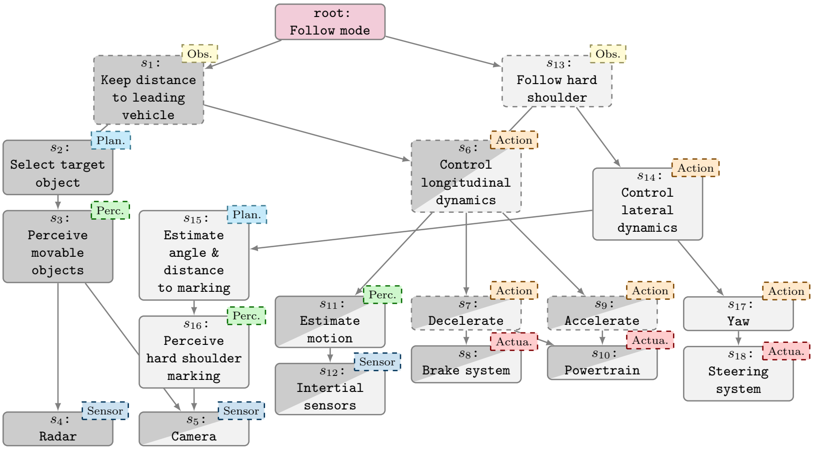

Fig. 6: Complete skill graph expressing an automated vehicle follow mode.

a framework for developing diagram editors in the context of model-driven development. The prototype allows practitioners to model and annotate well-formed skill graphs with safety guarantees as described in Section 3.

Thereupon, we implemented our compositional verification approach as described in Section 4. SKEDITOR allows to synthesize hybrid programs of specific skills with respect to their dependencies in the skill graph. Compliance checks of the provided safety guarantees are performed by employing the deductive theorem prover KEYMAERA X [14] in version 4.7.3. SKEDITOR and all experimental results can be found online.[3] We use the SKEDITOR to answer research questions *RQ-1* and *RQ-2*.

#### 5.2 Case Study: Vehicle Follow Mode

To illustrate the practicality of our approach, we model and verify the *vehicle follow mode* of an automated protective vehicle as adopted from Nolte et al. [26] and depicted in Figure 6. The aim of was to develop an unmanned protective vehicle which is able to drive on the hard shoulder autonomously (i.e., without any human interaction). On the lowest level, the skill graph consists of three sensors (i.e., ***Radar***, ***Camera***, and ***Inertial sensor***) to perceive information from the environment. Additionally, three actuators (i.e., ***Brake system***, ***Powertrain***, and ***Steering system***) represent concrete technical aspects. These skills propagate information about typical properties of a concrete model of a vehicle (e.g., the *maximal deceleration*). As highlighted with two shades of gray, this skill graph is dividable into two separate skill graphs, which we refer to as $G_1$ and $G_2$. $G_1$ has ***Keep distance to leading vehicle*** as the root skill, which is responsible for ensuring a minimum distance to a leading vehicle. $G_2$ has ***Follow hard shoulder*** as root skill, which is responsible for ensuring the vehicle's position inside the lane markings on a road. Skills shared by both skill graphs are highlighted with both shades.

[3] https://github.com/TUBS-ISF/Skeditor

| Skill | Requirement |
|---|---|
| *Follow hard shoulder* | Vehicle deviates from the center of the lane by at most half the lane width |
| *Control lateral dynamics* | Lateral controller must guarantee overshoot of less than 25 cm |
| *Yaw* | Vehicle yaw rate must not exceed $0.3\,\mathrm{rad\,s^{-1}}$ |
| *Control longitudinal dynamics* | Vehicle speed must not exceed $2.7\,\mathrm{m\,s^{-1}}$ |
| *Accelerate* | Acceleration must not exceed $4\,\mathrm{m\,s^{-2}}$ |
| *Decelerate* | Vehicle must at least provide a deceleration of $5\,\mathrm{m\,s^{-2}}$ |
| *Keep distance to leading vehicle* | Vehicle must keep a minimum distance of 10 m to leading vehicle |
| *Select target object* | Object recognition must always select an object of lateral position of $x > 10\,\mathrm{m}$ |
| *Perceive movable objects* | Object recognition must track vehicles of relative speeds between 0 and $60\,\mathrm{m\,s^{-1}}$ |
| *Estimate angle and distance to marking* | Angle to lanemarking must be extracted with maximum error of $\pm 0.5$ degrees and distance to lanemarking must be extracted with maximum error of $\pm 3$ cm |
| *Perceive hard shoulder* | Image processing must extract right edge of shoulder marking with a maximum error of 20 cm |
| *Estimate motion* | Vehicle velocity must be estimated with a maximal error of $\pm 0.03\,\mathrm{m\,s^{-1}}$ |

Table 2: Specified safety requirements for the vehicle follow mode as adopted from Nolte et al. [26].

The overall procedure ***Follow mode*** (i.e., the composition $G_1 \circ G_2$) requires a combination of autonomously following a leading vehicle (i.e., skill $s_1$) and following the lane marking (i.e., skill $s_{13}$). The informal safety guarantees for the skill graph of our case study are adopted from Nolte et al. [26] and illustrated in Table 2. Requirements are typically given informally, which is why we translated them to their formal counterpart. For our case study, we focus on four particular skills, as these are the only non-trivial skills in our case study that comprise both, the vehicle's dynamics and a control algorithm. Namely, these skills are ***Control longitudinal dynamics*** ($s_6$), ***Control lateral dynamics*** ($s_{14}$), ***Follow hard shoulder*** ($s_{13}$), and ***Keep distance to leading vehicle*** ($s_1$).

**Example 2** *Consider skill* **Control longitudinal dynamics** *($s_6$) in the context of the overall skill graph. Skill $s_6$ comprises the dynamic system for the longitudinal motion of the vehicle while depending on skills* **Accelerate** *and* **Decelerate** *as well as the perception skill* **Estimate motion**. *The* control algorithm *of this skill as part of the hybrid program complies with the safety requirements as given in Table 2 (e.g., velocity ($v_s$) must not exceed* $2.7\,\mathrm{m\,s^{-1}}$*). Preconditions for this skill are propagated from skills* **Estimate motion**, **Accelerate**, *and* **Decelerate**, *and guarantee that the vehicle provides a maximal deceleration of* $5\,\mathrm{m\,s^{-2}}$ *(B) and a maximal acceleration of* $4\,\mathrm{m\,s^{-2}}$ *(A). Table 3 summarizes all attributes of skill $s_6$.*

| | |
|---|---|
| $X_{\mathtt{def}} = \{x, v, v_{\mathtt{max}}\}$ | |
| $X_{\mathtt{req}} = \{a, A, B, ep, t\}$ | |
| $\alpha ::=$ | $\mathtt{init} \rightarrow [(ctrl; dyn)*](\mathtt{guar})$ <br> $\mathtt{init} \equiv v \geq 0 \wedge v \leq v_{\mathtt{max}} \wedge A > 0 \wedge A \leq 4 \wedge B \geq 5 \wedge v_{\mathtt{max}} = 2.7$ <br> $ctrl \equiv (?v_{\mathtt{max}} - v \leq \mathsf{margin}); a = *; -B \leq a \leq 0;$ <br> $\cup ?v_{\mathtt{max}} - v \geq \mathsf{margin}); a = *; -B \leq a \leq A; )$ <br> $\mathsf{margin} \equiv ep * A$ <br> $dyn \equiv t := 0; x' = v, v' = a, t' = 1 \& v \geq 0 \wedge t \leq ep$ <br> $\mathtt{guar} \equiv v \leq v_{\mathtt{max}}$ |
| $\tau = \mathtt{action}$ | |
| $\Phi = \{v_s \leq v_{\mathtt{max}}\ (2.7\,\mathrm{m\,s^{-1}})\}$ | |

Table 3: Attributes for skill ***Control longitudinal dynamics ($s_6$)***.

### 5.3 Results

All measurements were conducted on an Intel i7-6600U CPU @ 2.60GHz with 12 GB RAM and Z3 [12] in version 4.6.0 was used as the underlying solver for KeYmaera X in version 4.7.3.

***RQ-1: How does the skill-based methodology compare to monolithic modeling and verification?*** We modeled the overall behavior of $G_1 \circ G_2$ as a monolithic model (i.e., following the hard shoulder and following a leading vehicle in concert) as described in Section 4.2. As mentioned before, the skill-based approach has a high reuse potential. Each skill needs to be verified only once, and the verification results can be reused in other skill graphs (cf. Theorem 1). While in case of a change of parameters or an update of control algorithms the monolithic model has to be re-modeled and re-verified completely, a change impact analysis identifying only affected skills may reduce the re-verification effort even further for the compositional approach. Importantly, skill $s_{13}$ and the monolithic model could only be verified interactively, whereas skills $s_1$, $s_{14}$, and $s_6$ were verified fully automatically with the automatic proof search of KeYmaera X. Chances of an automatic re-verification are thus higher with the skill-based methodology.

An important hypothesis of ours is that skill-based verification is more effective in discovering modeling defects compared to a monolithic model. To get some insights into this hypothesis, we developed three initial experiments to render the verification attempt invalid. We (1) changed the safety guarantee of skills, (2) changed the control algorithm of skills, and (3) did a combination of both and compared these results to the same changes performed in the monolithic model. Following our methodology helped to trace and resolve defects effectively with respect to this case study, whereas identifying multiple modeling defects in the monolithic model became quickly intractable. During the resolution of Scenario 3, re-verification had to be performed several times for the monolithic model (i.e., resolving one conflict at a time), which emphasizes the advantage of our compositional approach over the monolithic modeling. However, we do not want to overclaim the importance of our insights, as more complex experiments

| | Verified Skill | | | | Proof steps | | | | | |
|---|---|---|---|---|---|---|---|---|---|---|
| | $s_1$ | $s_6$ | $s_{13}$ | $s_{14}$ | $s_1$ | $s_6$ | $s_{13}$ | $s_{14}$ | $\Sigma$ | $\Sigma_{\text{reuse}}$ |
| $G_1$ | ✓ | ✓ | | | 4,746 | 3,769 | | | 8,515 | 8,515 |
| $G_2$ | | ✓ | ✓ | ✓ | | 3,769 | 16,924 | 7,223 | 27,916 | 24,147 |
| $G_1 \circ G_2$ | ✓ | ✓ | ✓ | ✓ | 4,746 | 3,769 | 16,924 | 7,223 | 32,662 | 0* |
| $\Sigma_{\texttt{total}}$ | | | | | | | | | **69,093** | **32,662** |

*No re-verification of skills with Theorem 1

Table 4: Comparison of the verification effort for skill-based compositional verification.

and a larger evaluation have to be conducted to adequately test whether our hypothesis is significant.

***RQ-2: To what extent can skill-based compositional verification reduce the verification effort?*** To answer RQ-2, we measured the verification effort in proof steps for each of the three skills mentioned before per skill graph. In Table 4, we summarize the results. Column *Verified Skill* describes which skill is part of which skill graph and column *Proof steps* compares the number of proof steps needed for each skill individually. A common scenario is to model and verify each maneuver individually (i.e., each skill graph). The total verification effort $\Sigma_{\texttt{total}}$ would then cumulate to 69,093 proof steps. Instead, our skill-based approach allows to reuse verification results for skill $s_6$ in skill graph $G_2$ and per Theorem 1 even the verification results for all skills in skill graph $G_1 \circ G_2$. Entries highlighted in gray indicate that the respective skill could be reused instead of re-verification. The compositional approach needs approximately 53% less proof steps in our case study.

### 6 Related Work

***Skill Graphs.*** Maurer [23] pioneered the concept of skills by introducing so-called *abilities* in vehicle guidance systems. Abilities are similar to skills, as they concisely describe the capabilites of a vehicle, and are intended to be permanently monitored at run-time to enforce safety mechanics. Reschka et al. [32,33] introduced skill graphs informally in their work giving definitions for skills and abilities in relation to autonomous vehicles. Nolte et al. [26] built upon this approach by employing the informal concept of skill graphs for the development of self-aware automated road vehicles. We adopted their case study to evaluate our skill-based verification approach.

***Hybrid Systems and Verification of Cyber-Physical Systems.*** Hybrid systems [3] are a generalization of timed automata [4] and well-suited for modeling and verifying cyber-physical systems. Krishna et al. [20] show that using hybrid automata to model and verify cyber-physical systems is, in principle, feasible. Typically, hybrid systems are verified employing reachability analyses and model checking [2, 21, 22, 35]. However, these technqiues are not compositional in general (i.e., modular verification of individual parts to establish correctness of the entire systems is not possible). It is also not intended to generate and reuse proofs to increase trust in the system's correctness, as, for instance, possible with theorem proving. To address this issue, we built our methodology upon the notion of *hybrid programs* [30] and the theorem prover KeYmaera X [14], which helped us to also satisfy the important property of compositionality in the modeling and formal verification of hybrid systems. We further extend this concept with skill graphs by modularizing the verification of complex driving tasks, such that the verification of the entire behavior is reduced to simpler sub-tasks and compatibility checks.

Finally, there exists a seamless connection to the work conducted by Müller et al. [24], who present a compositional component-based approach for the verification of hybrid systems based on hybrid programs. Skill graphs provide an abstract and organized view of the system and are applied (1) in the verification and validation phase of the *requirements analysis* and (2) the early stages of the *design phase*. Subsequently, a skill may be implemented by a set of multiple interacting components to take more necessary specifics into account, such as communication protocols and resource consumption. To conclude, the process of refining skill graphs including their safety requirements to formally specified component-based systems exhibits a high level of quality assurance at the level of both, requirement engineers and software architects.

### 7 Conclusion and Future Work

In this work, we proposed skill-based verification of cyber-physical systems with the notion of skill graphs that (1) encourages the modular development of small and reusable actions in isolation, and (2) enables the identification of poorly defined requirements in early software development processes by considering formal verification of hybrid systems. We provide the first formalization of skill graphs, showed how skill graphs and hybrid programs can be combined, and also introduced a proved notion of compositionality for skills. The investigated case study on a *vehicle follow mode* showcases that the compositionality property of skill graphs is important for scaling, as the verification effort is reduced by more than 53%. Compositionality is particularly important for model and software evolution, as costly re-verification of a skill's requirement can be minimized.

For the future, we want to enable the composition of skills with dissimilar hybrid programs, for which the theoretical groundwork partially exists. Moreover, our current focus is on the integration of skill graphs into software engineering practices for cyber-physical systems to amplify the utilization of formal methods from the start of new software projects.

**Acknowledgements.** We are grateful to Enis Belli and Arne Windeler for their help with the implementation of Skeditor. This work was supported by the DFG (German Research Foundation) under the Researcher Unit FOR1800: Controlling Concurrent Change (CCC).
